# Eliminating hydrolytic activity without affecting the transglycosylation of a GH1 *β*-glucosidase

**DOI:** 10.1007/s00253-016-7833-9

**Published:** 2016-09-27

**Authors:** Pontus Lundemo, Eva Nordberg Karlsson, Patrick Adlercreutz

**Affiliations:** Department of Chemistry, Biotechnology, Lund University, P.O. Box 124, SE-221 00, Lund, Sweden

**Keywords:** *β*-glycosidase, Transglycosylation, pH-dependent enzyme mechanism

## Abstract

Unveiling the determinants for transferase and hydrolase activity in glycoside hydrolases would allow using their vast diversity for creating novel transglycosylases, thereby unlocking an extensive toolbox for carbohydrate chemists. Three different amino acid substitutions at position 220 of a GH1 *β*-glucosidase from *Thermotoga neapolitana* caused an increase of the ratio of transglycosylation to hydrolysis (*r*_s_/*r*_h_) from 0.33 to 1.45–2.71. Further increase in *r*_s_/*r*_h_ was achieved by modulation of pH of the reaction medium. The wild-type enzyme had a pH optimum for both hydrolysis and transglycosylation around 6 and reduced activity at higher pH. Interestingly, the mutants had constant transglycosylation activity over a broad pH range (5–10), while the hydrolytic activity was largely eliminated at pH 10. The results demonstrate that a combination of protein engineering and medium engineering can be used to eliminate the hydrolytic activity without affecting the transglycosylation activity of a glycoside hydrolase. The underlying factors for this success are pursued, and perturbations of the catalytic acid/base in combination with flexibility are shown to be important factors.

**Electronic supplementary material:**

The online version of this article (doi:10.1007/s00253-016-7833-9) contains supplementary material, which is available to authorized users.

## Introduction

**Fig. 1:**
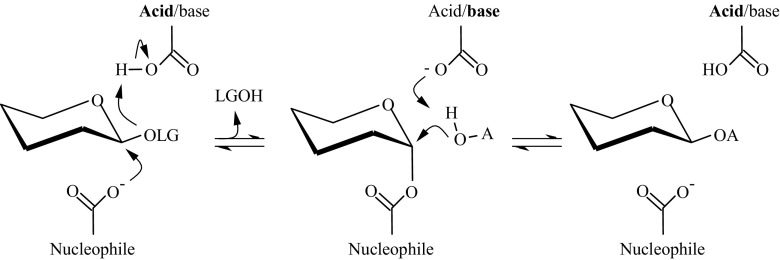
Reaction mechanism. The double displacement mechanism of retaining glycoside hydrolases. In this study, the leaving group (LG) is *p*-nitrophenol and the acceptor (A) is hexanol. The catalytic nucleophile is E164 and the catalytic acid/base is E349

Glycosylation is an important source of structural diversity of natural products. It can alter the properties of compounds in a multitude of ways, e.g., changing the flavor or smell (Ribeiro 2011; Roitner et al. 1984), improving water solubility (Chen et al. 2011) or stability (Yamamoto et al. 1990), or reducing skin irritation (Kurosu et al. 2002). Glycosylation can additionally be used to produce attractive biosurfactants, alkyl glycosides (van Rantwijk et al. 1999). In nature, glycosylation is mainly performed by Leloir glycosyltransferases. However, they are not well suited for glycosylation in vitro as they require expensive nucleotide-activated sugars as glycosyl donors and are often difficult to express. Glycoside hydrolases (GH) would be ideal enzymes for glycosylation with regard to their natural abundance, robustness, and wide acceptor specificity. For synthetic purposes, retaining GH are the most studied, since they all possess the machinery making transglycosidic activity possible. Most of them function via a double displacement mechanism, first proposed by Koshland (1953). In the first step, the catalytic nucleophile attacks the anomeric carbon of the glycosyl donor and forms a glycosyl enzyme intermediate, with the help of the catalytic acid that protonates the glycosidic oxygen (Fig. 1). Upon formation of the glycosyl enzyme, the pKa of the catalytic acid is reduced, allowing it to act as a general base in the second reaction step (McIntosh et al. 1996). The intermediate is subsequently deglycosylated by either water (hydrolysis) or another hydroxyl-containing acceptor (transglycosylation). Unfortunately, the use of GH for glycosylation is impaired by their naturally dominant hydrolytic activity.

A significant step toward utilizing GH for glycosylation was taken in 1998, with the development of the first glycosynthases (Mackenzie et al. 1998; Malet and Planas 1998). For retaining glycosynthases, the general strategy is to mutate the catalytic nucleophile, rendering the enzymes inactive. Using an activated donor, commonly glycosyl fluorides of the anomeric configuration opposite to the normal substrates, reactivates the enzyme. The glycosynthase no longer acts via a double-displacement mechanism but utilizes general base catalysis in combination with a good leaving group. With no glycosyl enzyme, hydrolysis can be avoided and the product cannot be hydrolyzed since it lacks good leaving group. However, glycosynthases do not always work efficiently and rely on activated glycosyl donors.

An alternative strategy to constructing glycosynthases is to limit the hydrolytic reaction of GH, transforming them into transglycosylases (TG). TG are rare in nature (Bissaro et al. 2015b; Larsbrink et al. 2012; Luang et al. 2013) but have been successfully produced from retaining GH through directed evolution (Bissaro et al. 2015a; Feng et al. 2005; Kone et al. 2009; Teze et al. 2014). Nevertheless, to be able to utilize the vast array of GH for creation of TG, we need to understand what governs their propensity for transglycosylation or hydrolysis.

From the numerous mutational studies aimed at improving the ratio of transglycosylation, three main mechanisms can be identified. The first mechanism is reducing the binding in glycone (−) subsites. Teze et al. improved disaccharide yield from 36 to 82 % through mutations in the −1 subsite (Teze et al. 2014). They hypothesized that the reduced interactions destabilize the transition states of the reaction, which affects hydrolysis more than transglycosylation and thereby improves the ratio of transglycosylation. The same effect has been seen in several other mutational studies (Arab-Jaziri et al. 2015; Aronson et al. 2006; Bissaro et al. 2015a; Feng et al. 2005).

The second common mechanism for improving transglycosylation is increasing the affinity in aglycone (+) subsites. Armand et al. (2001) found that several aromatic residues in the aglycone subsite of a GH10 xylanase were crucial for transglycosylation. The importance of aromatic residues in the aglycone subsite for transglycosylation has been seen also in GH16 and GH18 enzymes (Johansson et al. 2004; Taira et al. 2010). Moreover, Feng et al. (2005) reasoned that since deglycosylation is rate limiting, increased affinity of the acceptor would improve transglycosylation, which it is also found for a double mutant of a GH1 *β*-glucosidase. Higher ratios of transglycosylation have also been seen in other mutational studies with improved affinity in aglycone subsites (Champion et al. 2012; Lu et al. 2009; Osanjo et al. 2007).

The third popular strategy for promoting transglycosylation in GH is disrupting the binding of the catalytic water, which can be done in a multitude of ways. Honda et al. (2008) removed a known hydrogen-bonding interaction with the catalytic water and thereby reduced the hydrolytic reactivity of an inverting xylanase. Other studies have focused on improving the hydrophobicity of the entrance to the active site (Frutuoso and Marana 2013; Kuriki et al. 1996) or acceptor subsite (Lundemo et al. 2013). Natural TG could instead function through locking water molecules in hydrogen-bonding networks offset from the preferred position for a catalytic water, as demonstrated for a GH31 *α*-transglucosylase (Larsbrink et al. 2012).

Common in all studies mentioned above is that the reaction rate of the transglycosylation is reduced, although not as much as the hydrolysis. Even natural TG are commonly much less efficient catalysts than their hydrolytic counterparts (Bissaro et al. 2015b).

Apart from mutational studies, the propensity for transglycosylation has been affected through a variety of other methods. These methods include increasing reactant concentrations (Giacomini et al. 2002; Mala et al. 1999), changing reaction temperature (Ribeirao et al. 1997) or pH (Bonnin et al. 1997; Oikawa et al. 2001; Saitoh et al. 1995; Seidle and Huber 2005), adding cosolvent (Perez-Sanchez et al. 2011), and reducing water activity (Lundemo et al. 2014).

One of these methods showed that the hydrolytic activity of a *β*-glucosidase from *Aspergillus niger* could be effectively eliminated without affecting the rate of transglycosylation, through utilizing the enzymes’ seemingly constant transglycosylation rate across a wide pH range (Seidle and Huber 2005). Unfortunately, this property has not been found in any other glycoside hydrolase, to the best of our knowledge.

In this paper, we show that although transglycosylation is highly dependent on pH for the wild-type *β*-glucosidase from *Thermotoga neapolitana*, it can be changed by single mutations. The transglycosylation rate of three single mutant enzyme variants is unaffected at high pH, while the hydrolytic reaction is reduced. Subsequently, complete elimination of the hydrolytic side reaction and quantitative yields for our model reaction were made possible, without reducing the rate of transglycosylation. Furthermore, the molecular determinants for this success are pursued, in order to enable a novel strategy for improving the synthetic usefulness of glycosidases.

## Materials and methods

### Material

Hexyl-*β*-d-glucoside (HG), *p*-nitrophenol (*p*NP), and *p*-nitrophenol-*β*-d-glucoside (*p*NPG) were obtained from Sigma-Aldrich (St Louis, Missouri, USA) and all other chemicals from VWR International (Stockholm, Sweden).

### Mutagenesis

The gene encoding *Tn*Bgl1A was previously cloned into PET22b(+) (Novagen, Madison, WI, USA) (Turner et al. 2006). A reduced site saturation mutagenesis was generated using the QuikChange Multi Site-Directed Mutagenesis Kit (Stratagene, La Jolla, CA), using the sequence with GenBank accession number KC776911 as the template and the primer 5′-GGCAAAATTGGCATTGTGTTCNDTAATGGCTACTTCGAACCAGC-3′. The resulting plasmid library was retransformed into *Escherichia coli* Nova Blue cells for storage and into *E. coli* BL21 (Novagen, Madison, WI, USA) for expression. Functional mutants were selected from a 96-well assay, and the complete gene was sequenced by GATC Biotech AG (Konstanz, Germany) to confirm the mutations. Selected single mutants were produced in larger scale for characterization.

### Expression and purification

The enzymes were synthesized in 0.5-l cultivations of *E. coli* BL21 (Novagen, Madison, WI, USA) in Erlenmeyer flasks at 37 °C, pH 7, in Luria-Bertani (LB) media containing 100 μg/ml ampicillin and inoculated with 1 % overnight precultures. After reaching 0.6 in optical density, measured at 620 nm, *Tn*Bgl1A expression was induced by addition of 0.5 ml 100 mM isopropyl-*β*-d-1-thiogalactopyranoside (IPTG) and production was continued for 20 h. Cells were harvested by centrifugation for 10 min (4 °C, 5500×*g*), re-suspended in binding buffer (20 mM imidazole, 20 mM Tris-HCl, 0.75 M NaCl, pH 7.5), and lysed by sonication 6 × 3 min at 60 % amplitude and a cycle of 0.5 using a 14-mm titanium probe (UP400 S, Dr. Hielscher, Teltow, Germany). Heat treatment (70 °C, 30 min) and centrifugation (30 min, 4 °C, 15,000×*g*) were used to remove most of the native *E. coli* proteins before purification by immobilized metal affinity chromatography using an ÄKTA prime system (Amersham Biosciences, Uppsala, Sweden). The protein slurry was applied to a HisTrap FF crude column (GE Healthcare, Uppsala, Sweden) pretreated with 0.1 M copper(II) sulfate. Bound proteins were eluted using elution buffer (250 mM imidazole, 20 mM Tris-HCl, 0.75 M NaCl, pH 7.5). Fractions containing protein were pooled and dialyzed against 50 mM citrate phosphate buffer, pH 5.6, overnight using a 3500-Da molecular weight cutoff dialysis membrane (Spectrum Laboratories, Rancho Dominguez, CA, USA) and stored at −20 °C until use. Purity of the expressed proteins was estimated using SDS-PAGE according to Laemmli (1970).

### Water activity control

Substrate solutions (34 mM *p*NPG in hexanol) were incubated over saturated salt solutions to define water activities. The salts used for equilibration were KCH_3_CO_2_ (*a*_w_ = 0.23), MgCl_2_ (*a*_w_ = 0.33), Mg(NO_3_)_2_ (*a*_w_ = 0.53), NaCl (*a*_w_ = 0.75), KCl (*a*_w_ = 0.84), and K_2_SO_4_ (*a*_w_ = 0.97) (Greenspan 1977) (Goderis et al. 1987). Triplicate samples from each equilibrated hexanol sample were injected on an 899 Karl Fischer coulometer (Metrohm, Herisau, Switzerland). The obtained relation between water activity and water amount was used to estimate water activity in the transferase reactions.

### Transglycosylation in monophasic hexanol

Reactions of 2 ml 34 mM *p*NPG were started and concurrently set to the desired water activity by addition of 2–20 ng enzyme in a determined volume of 0.1 M citrate phosphate buffer, pH 5.6, based on the abovementioned Karl-Fisher calibration curve. The reactions were kept in a ThermoMixer (HLC Biotech, Bovenden, Germany) set to 70 °C and 700 rpm.

### Buffers used

The buffers used in this study are 0.1 M of the following, unless otherwise stated: citrate phosphate (pH 3.0–6.0), sodium phosphate (pH 6.0–8.5), Tris-HCl (pH 8.0–9.0), glycine-NaOH (pH 9.0–10.5), and phosphate-NaOH (pH 11.0–11.5).

### Biphasic transglycosylation

In a ThermoMixer (HLC Biotech, Bovenden, Germany) set to 70 °C and 800 rpm, 2550 μl 34 mM *p*NPG in water-saturated *n*-hexanol was preheated. Reactions were started by addition of 450 μl enzyme (≈10 ng/ml) in 0.1 M citrate phosphate buffer, pH 5.5.

### Steady-state kinetics for hydrolysis

In 96-well PCR plates, each enzyme variant was assayed at nine different pHs using seven different concentrations of *p*NPG with triplicate samples for each concentration. On ice, 195-μl *p*NPG solution was added to each well and 5-μl enzyme solution (0.67 ng/ml). Triplicate *p*NP standards were loaded on each plate as well as blanks for each combination of pH and *p*NPG concentration, to account for spontaneous hydrolysis. The reactions were started by moving the plate to a preheated thermomixer set to 70 °C and 300 rpm mixing for a set time, cooling on ice for 5 min before transferring 40 μl to a flat-bottomed 96-well plate, and reading the absorbance at 400 nm using a Multiskan GO plate reader (Thermo Scientific, USA). Two replicates were run for each plate to ensure keeping in the linear range. The incubation times were 10 and 30 min for low pH, 10 and 20 min for medium pH, and 5 and 10 min for high pH plates due to the higher absorbance of *p*NP in its deprotonated state.

### HPLC analysis

Samples were withdrawn from the hexanol phase through a septum lid to follow the reaction by analysis on HPLC. Transferase and reverse hydrolysis reactions were monitored using reverse-phase HPLC (L-7100 pump, L-7000 interface, L-7250 autosampler with a 20-ml injection loop, and L-7400 UV detector; LaChrom; Hitachi Ltd., Tokyo, Japan) with the HPLC equipped with an evaporative light-scattering detector (500 ELSD; Alltech Associates Inc., Deerfield, IL) with an evaporator temperature of 94 °C and a nebulizer gas flow of 2.5 standard liters per minute and a Kromasil 100-5C18 column (4.6 μm by 250 mm; Kromasil; Eka Chemicals AB, Separation Products, Bohus, Sweden). A gradient was applied from 50 to 70 % methanol in Milli-Q water over 5 min and kept at 70 % methanol for 1 min before returning to initial conditions for reequilibration. A constant flow rate of 1.0 ml/min was used. *p*NPG elutes after 3.5 min and was followed with the ELSD detector. HG and *p*NP both have a retention time of 7.5 min, but HG does not absorb at 405 nm and *p*NP is too volatile to be detected by the ELSD detector. Sample chromatograms can be found in Supplementary Fig. S1. Concentrations were determined by use of eight-point external standard curves. Sampling was done only from the hexanol phase, and partitioning to the aqueous phase was accounted for by using experimentally determined apparent partitioning coefficients (Lundemo et al. 2013). After compensating for withdrawn sample volume, the total specific initial reaction rate and the ratio of transglycosylation over hydrolysis (*r*_s_/*r*_h_) were calculated. The synthetic reaction rate equals the formation of HG, and the hydrolytic reaction rate is calculated by subtracting the HG formation rate from the total reaction rate (formation rate of *p*NP). No self-condensation of *p*NPG was observed at the *p*NPG concentrations used.

### Molecular dynamic simulation

As a starting point for the simulations, the coordinates from the crystal structure of *Tn*Bgl1A were used (PDB code 5IDI). Point mutations were introduced using Scwrl4 (Krivov et al. 2009). The molecular dynamics (MD) simulation was performed using GROMACS 5.0 (Pronk et al. 2013) and the OPLS-AA force field (Kaminski et al. 2001) at 343 K and with ionization states corresponding to pH 6 and pH 10, predicted by PROPKA 3.1 (Olsson et al. 2011). The enzyme variants were placed in the center of a cubic box filled with 21,289–21,750 water molecules from a simple point charge water model (Hermans et al. 1984). Before MD, the system was energy minimized and equilibrated with constant volume and temperature followed by constant pressure and temperature. Simulations of 500 ps were performed with a time step of 2 fs, and the coordinates were saved every 5000 steps (10 ps).

## Results

**Fig. 2:**
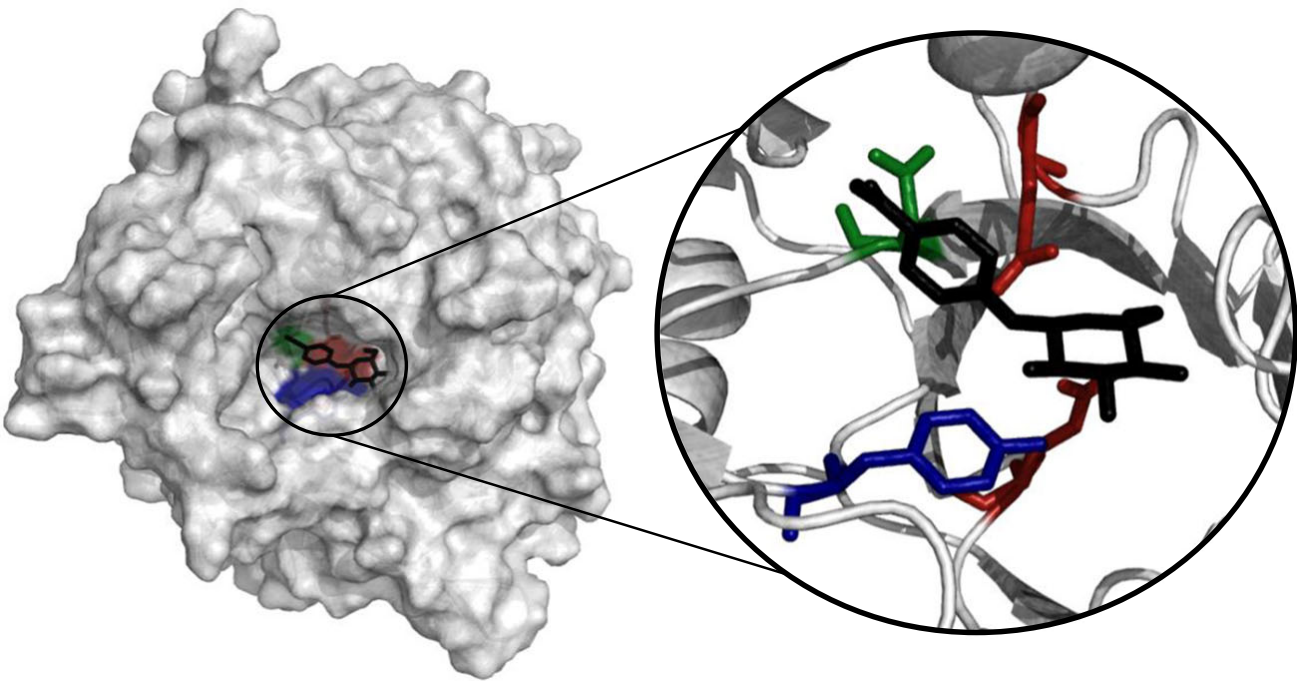
Active site. Position of N220 (*green*) in *Tn*Bgl1A in relation to the catalytic acid/base E168 (*top*, *red*) and nucleophile E349 (*bottom*, *red*), position Y293 (*blue*), and a docked *p*NPG molecule (*black*) (Color figure online)

In a previous study, a single mutation, N220F (Fig. 2), with substantial effect on the ratio of transglycosylation over hydrolysis was identified (Lundemo et al. 2013). N220 is positioned in the second layer of subsite +1, and the homologous position in Zm*Bgl*1 has a water-mediated interaction to the bound oligosaccharides (Chuenchor et al. 2011). This position was investigated in detail in this study with the aim of understanding the reason for its importance and evaluating whether further improvements to the ratio of transglycosylation are possible.

### Improved transglycosylation of mutants at position N220

A reduced site saturation library for position 220 was generated, and a selected group was further characterized. Specifically, one small nucleophilic (Cys, C), one small hydrophobic (Leu, L), an acidic (Asp, D), and a basic (Arg, R) along with two aromatic (Phe, F, and Tyr, Y) amino acids were selected.

To study transglycosylation, aqueous enzyme solution was added to *p*NPG in water-saturated hexanol, creating a two-phase system. As seen in Table 1, at optimal pH for total activity (pH 5.5), N220L lowered the reaction rate and specificity for transglycosylation significantly, while all other mutations improved the ratio of synthetic over hydrolytic activity (*r*_s_/*r*_h_). The positive effects on specificity for a hydrophilic amino acid, arginine, in Table 1 suggest that merely making the acceptor subsite more hydrophobic is not the only way to favor transglycosylation and warrants further investigation.

**Table 1:** Total activity and transglycosylation rate over hydrolysis rate (*r*_s_/*r*_h_) for variants of *Tn*Bgl1A run in a hexanol-water two-phase system at pH 5.5 and pH 10

Enzyme	Total initial reaction rate^a^ (μmol min^−1^ mg^−1^)		*r*_s_/*r*_h_^a^	
	pH 5.5	pH 10	pH 5.5	pH 10
*Tn*Bgl1Awt	224 ± 2	168 ± 1	0.33 ± 0.00	0.38 ± 0.00
N220C	260 ± 8	ND	0.73 ± 0.01	ND
N220L	1.4 ± 0.0	ND	0.12 ± 0.17	ND
N220D	256 ± 5	ND	0.41 ± 0.01	ND
N220R	265 ± 2	211 ± 15	2.21 ± 0.17	4.8 ± 0.9
N220F	134 ± 2	72 ± 1	1.45 ± 0.48	38 ± 14
N220Y	138 ± 2	113 ± 6	2.71 ± 0.21	8.3 ± 0.9

^a^Average ± standard deviation

### Influence of pH on transglycosylation and hydrolysis in hexanol/water

**Fig. 3:**
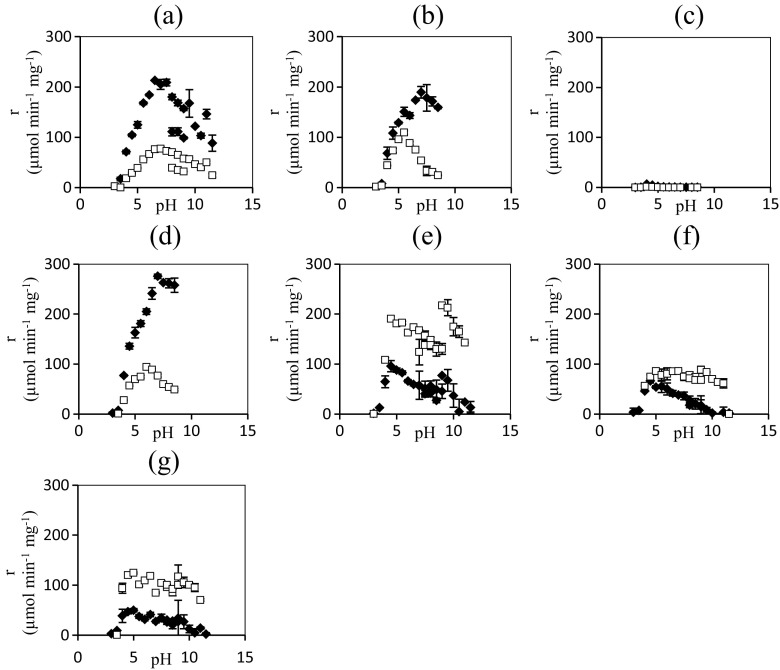
pH dependence of transglycosylation and hydrolysis. Specific initial reaction rate (*r*) for hydrolysis (*diamonds*) and transglycosylation (*squares*) for *Tn*Bgl1A in 85 % hexanol 15 % 0.1 M buffer systems with p-nitrophenyl-β-d-glucopyranoside as glycosyl donor. Alongside the wild-type enzyme (**a**), N220C (**b**), N220L (**c**), N220D (**d**), N220R (**e**), N220F (**f**), and N220Y (**g**) variants are presented. *Error bars* represent 1*σ*

For each enzyme variant, the two-phase reaction system was run at 18 different pH values and the total initial reaction rates of the hydrolytic and transglycosidic reactions were measured and are presented in Fig. 3. For the wild-type, N220C and N220D mutants, the hydrolytic activity dominates across the pH spectrum and the activity profiles have the expected bell shape. Interestingly, the most beneficial mutants (N220Y, N220F, and, to some extent, N220R) only display a strong dependence on pH for the hydrolytic reaction, while the transglycosylation is largely unaffected. This leads to improved *r*_s_/*r*_h_ with increased pH, and the effect persists until no hydrolytic side reaction is observed, similar to the results by Seidle and Huber (2005).

**Fig. 4:**
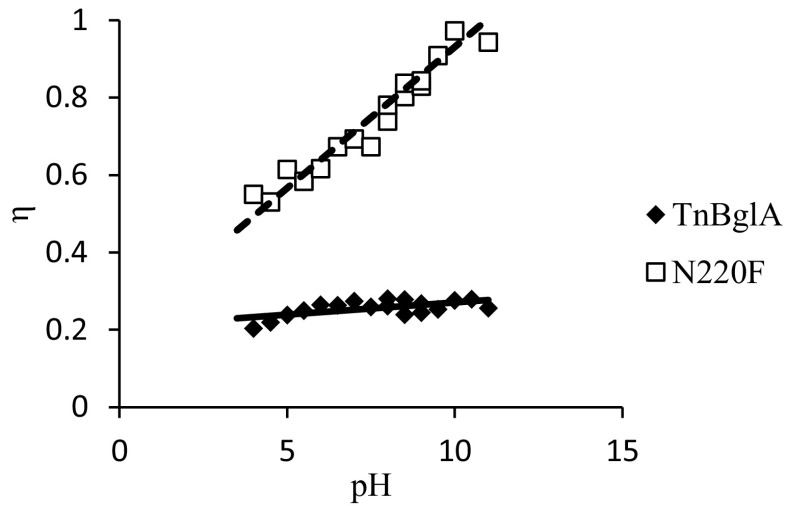
Comparison of wild type and mutant. The ratio of the transglycosylation rate and the total rate of substrate conversion, i.e., estimated yield (*η*) of *Tn*Bgl1A and mutant N220F as a function of pH in 85 % hexanol 15 % 0.1 M buffer systems with *p*-nitrophenyl-*β*-d-glucopyranoside as glycosyl donor

In the practical application of enzymatic reactions, the fraction of the substrate being converted to the desired product is of crucial importance. When using glycosidases for the synthesis of alkyl glycosides, the competition between transglycosylation and hydrolysis reactions is the key issue. To illustrate the influence of changes in the enzyme and modification of the reaction conditions, the ratio of the transglycosylation rate and the total rate of substrate (*p*NPG) conversion (*η*) were plotted as a function of pH for the wild-type enzyme and the N220F mutant (Fig. 4). In absence of secondary hydrolysis, *η* is a good estimate of the reaction yield. Secondary hydrolysis occurs at a rate of less than 1 % compared to the total reaction rate of all enzyme variants in this study (data not shown). Hence, it is obvious that the combination of a single mutation and a change in reaction pH is enough to provide an excellent catalyst for the transglycosylation reaction with a minimum of substrate being lost in the hydrolytic side reaction. In an attempt to uncover the mechanistic reason for this favorable result and thereby unlock a novel strategy for the construction of non-Leloir glycosyltransferases, a battery of additional tests was performed.

### Kinetics of hydrolysis in water

The two most prevalent hypotheses for improved ratio of transglycosylation in GH are reduced affinity in glycone-binding sites and improved affinity in aglycone-binding sites (Bissaro et al. 2015b). To acquire information on the binding of the glycone to the enzyme variants, a simplified system, without hexanol phase, was studied and the kinetic constants for *p*NPG hydrolysis were determined. Few significant effects of the mutations were seen, only the inactivation of N220L and the increased *k*_cat_ and *K*_M_ of mutant N220R (Supplementary Fig. S2). Interestingly, the latter effect is not seen for the other two mutants with large effects on specificity for transglycosylation (N220Y and N220F).

### Probing the ionization state of catalytic residues using hydrolysis kinetics in water

Further information on the reaction mechanism can be obtained from studies of the pH dependence of kinetic constants for hydrolysis. In a somewhat simplified model, the variation of *k*_cat_/*K*_M_ with pH can be described by the following equation (Cornish-Bowden 2012):

$$ \frac{V_{max}}{K_M}=\frac{{\left(\frac{V_{max}}{K_M}\right)}_{max}}{1+\frac{10^{-pH}}{10^{-p{K}_{E1}}}+\frac{10^{-p{K}_{E2}}}{10^{-pH}}} $$

The pKa values pK_E1_ and pK_E2_ are assumed to reflect the ionization of the nucleophile and the catalytic acid/base, an assumption supported by NMR for another GH by Joshi et al. (2000, 2001). All mutations lead to more narrow pH optima (seen as the difference between pK_E1_ and pK_E2_), but, in general, there were no significant changes to the optimal pH, with the exception of N220D and N220Y (Table 2, Fig. 6, Supplementary Fig. S3). Both show significant perturbation to the pKa of the nucleophile (+1.1 and +1.0 pH units, respectively), while N220Y additionally affected the pKa of the catalytic acid/base (+0.5 pH units). At pH values above 9, very low activity was observed for all enzyme variants in this aqueous system.

**Table 2:** Apparent pKa values of nucleophile (pK_E1_) and acid/base (pK_E2_) as obtained from the dependence of *k*_cat_/*K*_M_ on pH, presented with pH optimum and relative *k*_cat_/*K*_M_ at pH optimum

	pK_E1_^a^	pK_E2_^a^	pH_opt_	ΔpH_opt_	Relative *k*_cat_/*K*_M_
*Tn*Bgl1Awt	4.5 ± 0.2	6.5 ± 0.2	5.5	±0.0	1.0
N220C	5.0 ± 0.7	6.4 ± 0.7	5.7	+0.2	1.3
N220L	4.8 ± 0.5	5.7 ± 0.5	5.2	-0.3	0.0
N220D	5.6 ± 0.7	6.6 ± 0.7	6.1	+0.6	2.2
N220R	4.7 ± 0.5	6.1 ± 0.5	5.4	-0.1	2.5
N220F	4.6 ± 0.2	6.3 ± 0.2	5.4	-0.1	1.8
N220Y	5.5 ± 0.3	7.0 ± 0.3	6.3	+0.8	1.4

^a^Average ± st dev

### Investigating flexibility through molecular dynamic simulation

To investigate if the point mutations influence the overall flexibility of the enzyme, 500-ns MD simulations were performed under standard state conditions. The root-mean-square fluctuations of wild-type and N220R, N220F, and N220Y are presented in Supplementary Fig. S4. No significant changes in protein flexibility were observed. However, distortions of the catalytic acid/base were observed for the mutants N220Y and N220R after 50- to 500-ns simulation (Supplementary Fig. S5).

### The role of water for performance of enzyme variants in monophasic hexanol

**Fig. 5:**
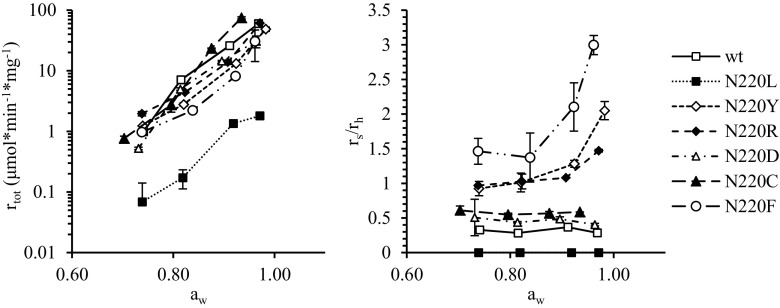
Water activity dependence. Total specific initial reaction rate (*left*) and transglycosylation (*r*_s_) over hydrolysis (*r*_h_) ratio (*right*) for *Tn*Bgl1A and single mutation variants of the same, in micro-aqueous hexanol at varying water activities (*a*_w_). *Error bars* represent 1*σ*

To further study the possibilities to influence the competition between transglycosylation and hydrolysis, experiments were carried out using hexanol as reaction medium and with the water activity being controlled by equilibration with saturated salt solutions. Figure 5 shows that all enzyme variants display exponential increase of reaction rates with increasing water activity, consistent with previous studies (Hansson et al. 2001; Lundemo et al. 2014; Mladenoska et al. 2007; Mladenoska et al. 2008). Moreover, in agreement with the two-phase system, N220L was significantly slower than the other mutants. For the wild-type enzyme and the N220L, N220D, and N220C, the ratio of transglycosylation to hydrolysis (*r*_s_/*r*_h_) was largely unaffected by water content, while this ratio increased with increasing water activity for the mutants N220R, N220F, and N220Y. This counter intuitive effect of water on *r*_s_/*r*_h_ has been observed previously for several GH (Hansson et al. 2001; Lundemo et al. 2014; Mladenoska et al. 2007; Mladenoska et al. 2008). The N220F variant reached a higher *r*_s_/*r*_h_ in this system without a macroscopic aqueous phase than in the two-phase system (Table 1), while the two-phase system gave the highest *r*_s_/*r*_h_ for N220R and N220Y.

## Discussion

To be able to utilize the vast array of GH for creation of TG, we need to understand what governs their predisposition for transglycosylation or hydrolysis. We show that a single point mutation in the second layer of the aglycone subsite of a *β*-glucosidase from *T. neapolitana*, in combination with high pH, can reduce the hydrolytic reaction to undetectable levels, hence transforming the enzyme into a transglycosylase. Moreover, in contrast to previously published studies, the reaction rates of the transglycosylation for these three mutants are maintained. Furthermore, in an attempt to unveil the mechanism behind this success, several factors influencing the propensity for transglycosylation have been studied.

### Alterations to substrate affinity

Kinetic evaluation of *p*NPG hydrolysis in aqueous media for *Tn*Bgl1A variants revealed no indication of improved or reduced affinity for the glycosyl donor, with the exception of variant N220R. This enzyme variant displayed an equal increase in *K*_M_ and *k*_cat_. An increased *K*_M_ suggests a decreased affinity for *p*NPG. Decreased affinity in subsite −1 affecting the glycosyl donor has been identified as a key factor for converting GH into TG (Bissaro et al. 2015b). However, position N220 is more likely to affect the aglycone (+1) subsite due to its position in the second layer of this subsite. Furthermore, the absence of an effect for the other two beneficial mutations (N220F and N220R) suggests that this is not the reason for the improved specificity for transglycosylation in this case.

### The role of the catalytic acid/base

MD simulations suggest a distortion of the catalytic acid/base for mutants N220Y and N220R after 50- to 500-ns simulation, while no such distortion is seen for wild-type *Tn*Bgl1A (Supplementary Fig. S5). This observation can be relevant for different reasons. Firstly, incorrect positioning of the catalytic base would hamper the deglycosylation step and thereby increase the lifetime of the glycosyl-enzyme intermediate. Extending the lifetime of the intermediate complex allows more time for the slower diffusing preferred acceptor, hexanol, to enter the active site. Alternatively, as described for a GH13 CGTase by Kelly et al. (2008), the catalytic conformation could be regained through acceptor binding, thereby making the enzyme unable to use water as acceptor. An alternative explanation is related to the catalytic water. The only non-water-mediated interaction to the potential catalytic water in the crystal structure of *Tn*Bgl1A is the acid/base. Distorting the acid/base could therefore lock the catalytic water in a position unfavorable for hydrolysis, similar to the proposed mechanism of natural TG (Larsbrink et al. 2012).

### Influence of protein flexibility

Glycosyltransferases are more flexible than GH, indicating that flexibility could be important for transglycosylation (Rojas-Cervellera et al. 2013). In addition, conformational changes have been proposed in the transglycosylation mechanism of both CGTases (Kelly et al. 2008; Uitdehaag et al. 1999) and glucanotransferases (Barends et al. 2007; Kaper et al. 2007). No significant changes in enzyme flexibility due to amino acid substitutions were observed in MD simulations (Supplementary Fig. S4). However, for the enzyme variants having the most promising transglycosylation activities, *r*_s_/*r*_h_ increased with increasing water activity. This suggests that flexibility indeed plays a role also for GH, since water is well established as molecular lubricant increasing the internal flexibility of enzymes (Affleck et al. 1992; Broos et al. 1995; Soares et al. 2003).

### Ionization state of the active site residues

**Fig. 6:**
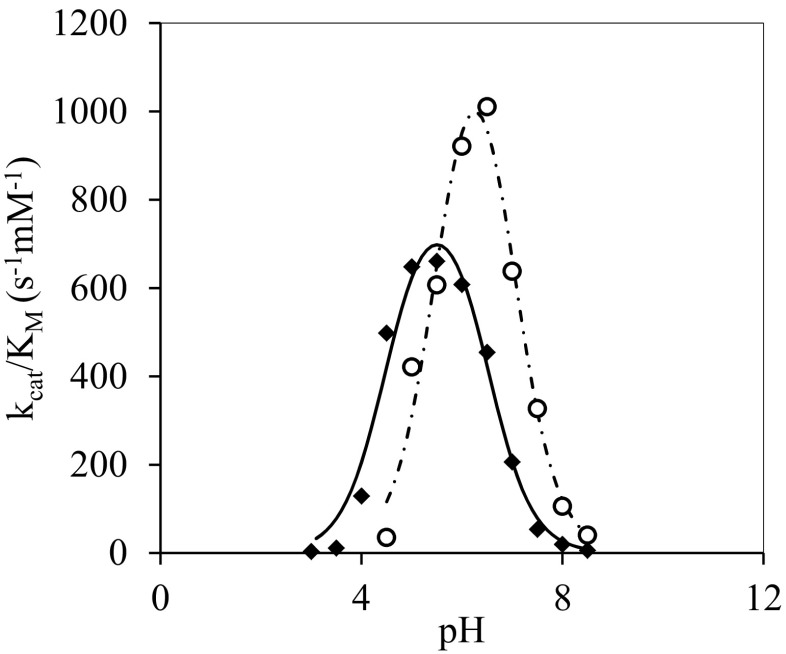
pH dependence of hydrolysis in water. pH dependence of *k*_cat_/*K*_M_ for *Tn*Bgl1A (*diamonds*) and N220Y mutant (*circles*) along with their model-fitted curves, *solid* and *dashed lines*, respectively

N220Y displays significantly altered pKa values for both nucleophile (pK_E1_) and acid/base (pK_E2_), as emphasized in Fig. 6, while N220R and N220F do not. Either the three mutations successfully improve *r*_s_/*r*_h_ through different mechanisms, or alternatively, the pKa shifts are of little importance for the improved specificity for transglycosylation and are merely a coincidence. Nevertheless, ionization of the catalytic residues plays a key role in the hydrolytic activity of the enzyme. For all enzyme variants, the hydrolytic activity is reduced at elevated pH, supposedly due to the catalytic acid losing the protonated state required for catalysis. Why this does not influence the transglycosylation is yet to be determined. Alternatively, the reduction of activity at high pH could be caused by Tyr293 (Fig. 2), losing its protonated state. This tyrosine has been suggested to stabilize the ionized state of the catalytic nucleophile and thereby facilitate the deglycosylation through acid catalysis (Seidle and Huber 2005). A stronger nucleophile acceptor may not require this acid catalysis. Furthermore, several mutational studies have indicated the involvement of this tyrosine in modulating the preference for transglycosylation (Bissaro et al. 2015a; Teze et al. 2014).

In conclusion, we successfully reduced the unwanted hydrolytic activity of a family 1 glycoside hydrolase from *T. neapolitana* to an undetectable level, without diminishing the efficiency for transglycosylation. This was achieved through single point mutations (N220F, N220R or N220Y), in combination with catalysis at a pH well above the hydrolytic optima. Moreover, the mechanism behind the efficiency of the mutation as well as the dependence on pH was investigated. MD simulations suggest a distortion of the catalytic acid/base for the mutants, which is not only a catalytic residue but also important for directing the catalytic water. In addition, the hydrolytic activity is diminished at high pH due to deprotonation of the catalytic acid, while the transglycosylation is retained. This suggests that transglycosylation with hexanol as acceptor is not dependent on a catalytic acid/base, the mechanistic reason for which is still unclear. Further studies are required to increase the molecular understanding of this phenomenon, to allow general applicability of this novel strategy for the generation of non-Leloir glycosyltransferases.

## Electronic supplementary material

ESM 1 (PDF 447 kb)
