# Supplementary material for: Eliminating hydrolytic activity without affecting the transglycosylation of a GH1 β-glucosidase
Source: Appl Microbiol Biotechnol. 2016 Sep 27;101(3):1121–31. doi: 10.1007/s00253-016-7833-9 (PMC5247548; doi:10.1007/s00253-016-7833-9)
Supplement: Supplementary file 1 — (PDF 447 kb) [file 253_2016_7833_MOESM1_ESM.pdf]

Supplementary material

Applied Microbiology and Biotechnology

Eliminating hydrolytic activity without affecting the transglycosylation of a GH1 *β*-glucosidase

Pontus Lundemo, Eva Nordberg Karlsson, Patrick Adlercreutz

Lund University, Dept of Chemistry, Biotechnology, P.O. Box 124, SE-221 00 Lund, Sweden

Corresponding author: Patrick Adlercreutz, Phone: +46462224842, Email: patrick.adlercreutz@biotek.lu.se

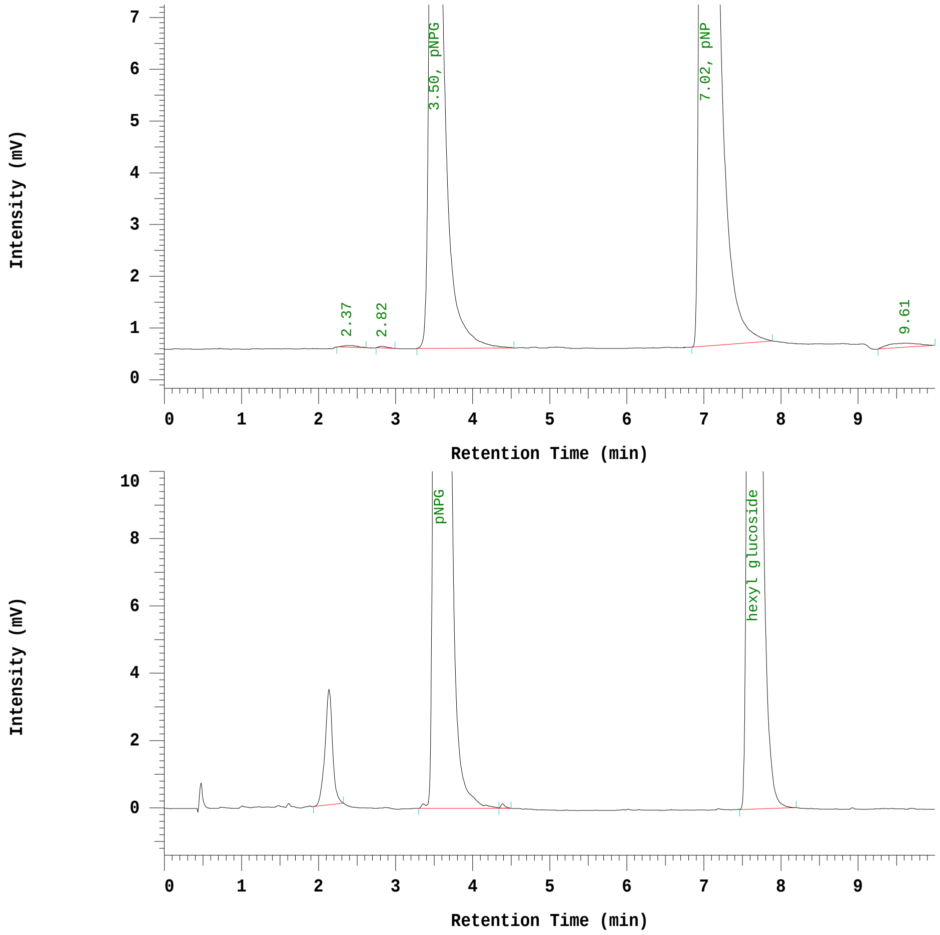

Fig. S1 Sample chromatograms of a transglycosylation reaction between *p*-nitrophenol-*β*-D-glucoside (*p*NPG) and hexanol. Absorbtion at 405nm (top) and ELSD detection (bottom) is used and the ELSD peak at 2.2 min is the void peak.

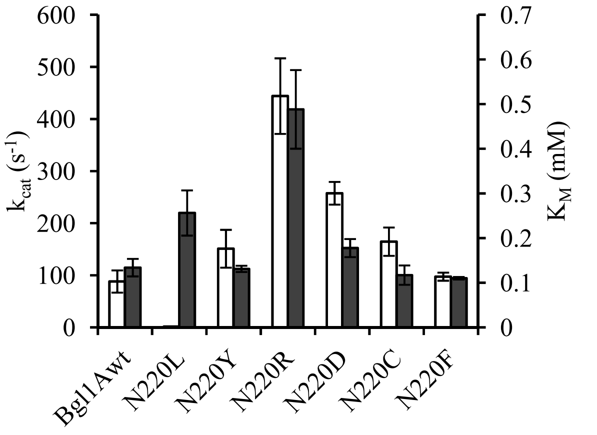

Fig. S2 Steady-state kinetic parameters, k_{cat} (white bars) and K_M (grey bars) for hydrolysis of *p*-nitrophenyl-*β*-D-glucopyranoside by *Tn*Bgl1A and single point mutants thereof in 0.1 M citrate phosphate buffer pH 6.0. Error bars represent 1σ.

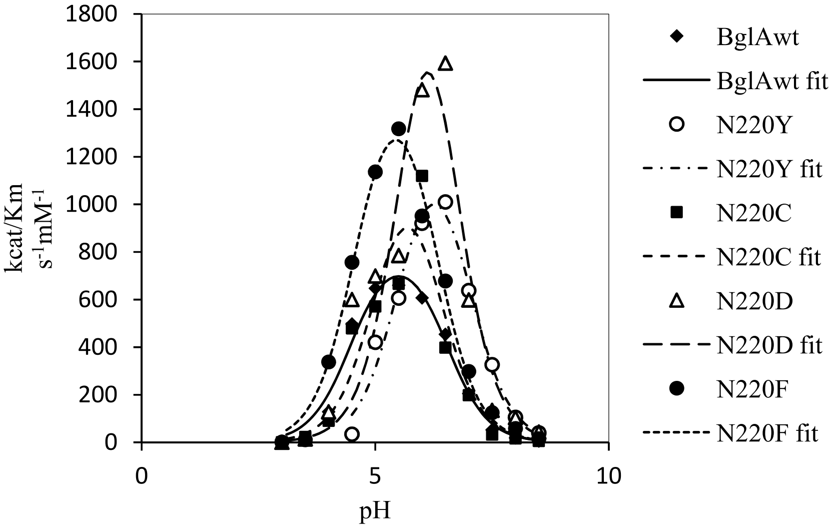

Fig. S3 pH dependence of k_{cat}/K_M for TnBgl1A and single mutants thereof along with their model-fitted curves.

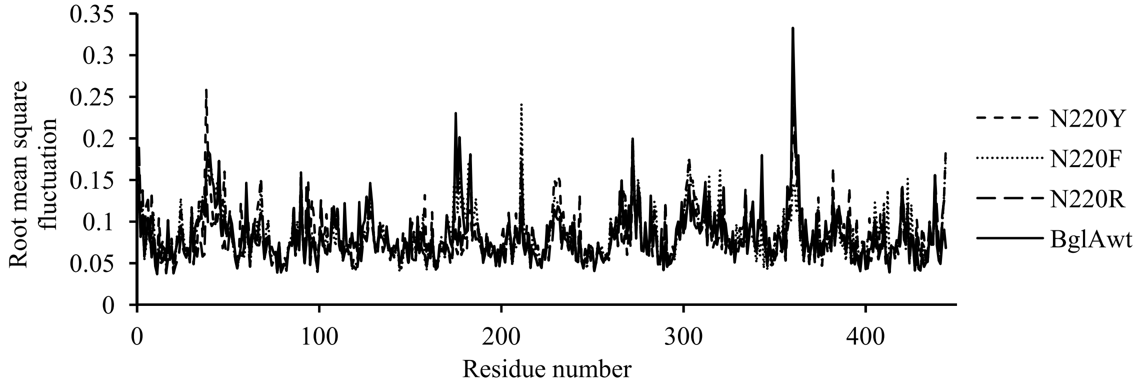

Fig. S4 Root mean square fluctuation for each amino acid of 500 ns simulations at room temperature for *Tn*Bgl1Awt and mutants N220Y, N220F and N220R.

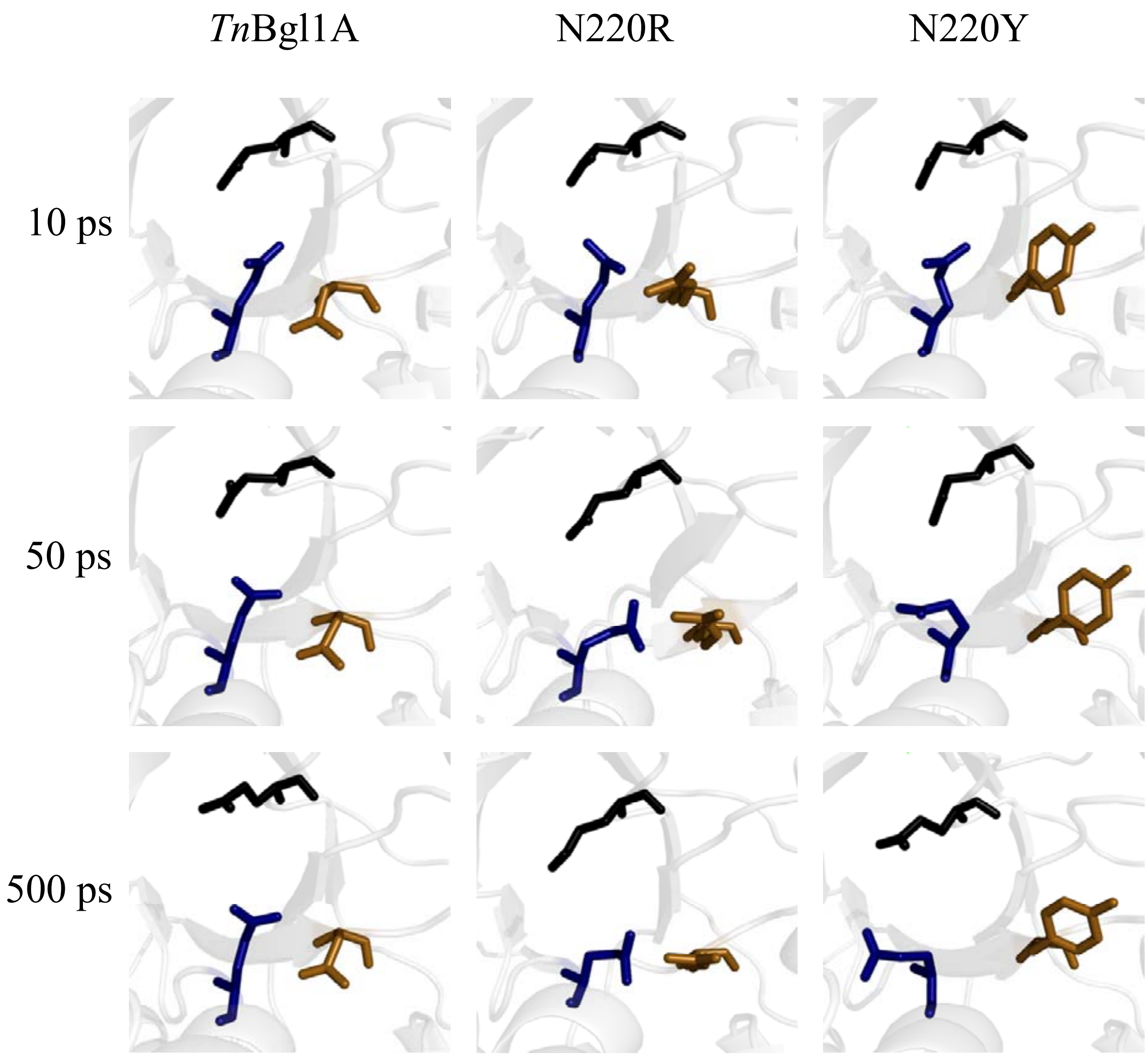

Figure S5 Orientation of the catalytic nucleophile (black), acid/base (dark blue) and position N220 (brown) at different time-points of a molecular dynamics simulation.
